# Elements of Chemistry and Dental Materia Medica

**Published:** 1893-06

**Authors:** 


### Bibliography.

ELEMENTS OF CHEMISTRY AND DENTAL MATERIA MEDICA. By J. C. Cassidy, D.D.S., M.D., Professor of Chemistry and Materia Medica in Ohio College of Dental Surgery. Cincinnati: Robert Clark & Co., 1893.

This work was designed by the author to test "the comparative value to dental students of the two approved didactic methods of teaching chemistry and materia medica,—*i.e.*, by lectures and quiz and by recitations from approved text-books, in connection with suggestive experiments."

This effort to combine chemistry and materia medica in one volume has the merit of novelty, and would seem, upon a theoretical view, a natural course of study. The difficulty has been, and, it is presumed, will always exist, to combine two very extended subjects within the compass of a small book and at the same time satisfy the critical mind. The author has performed his work in this instance with a considerable degree of success and with a minimum amount of error, but the student will, it is feared, find the necessary condensation, especially in the part devoted to materia medica, by no means equal to his needs. The author seems to have regarded this as a possible objection, for he says, in his preface, that "It may appear on first sight that our materia medica is too limited and overshadowed by the governing science of chemistry, but the combination of these two branches of our curriculum . . . necessarily prevents the complete exposition of at least those drugs which are unessential in dental practice."

The question of essentials in practice must necessarily be left to each individual teacher, but this branch, it should be remembered, has developed to such an extent in dentistry of recent years that its combination with that of chemistry, in the limits of this volume, cannot give altogether satisfactory results.

The plan of the author, it must be conceded, has a decided value if it be possible to extend it within the limits of a text-book. It is no disparagement, therefore, to say that while this effort does not entirely meet the needs of the student in its present form, a future edition may result in a work free from all objections, and prove of great value in the work of the schools.

